# Variation in clutch size in relation to nest size in birds

**DOI:** 10.1002/ece3.1189

**Published:** 2014-09-02

**Authors:** Anders P Møller, Frank Adriaensen, Alexandr Artemyev, Jerzy Bańbura, Emilio Barba, Clotilde Biard, Jacques Blondel, Zihad Bouslama, Jean-Charles Bouvier, Jordi Camprodon, Francesco Cecere, Anne Charmantier, Motti Charter, Mariusz Cichoń, Camillo Cusimano, Dorota Czeszczewik, Virginie Demeyrier, Blandine Doligez, Claire Doutrelant, Anna Dubiec, Marcel Eens, Tapio Eeva, Bruno Faivre, Peter N Ferns, Jukka T Forsman, Eduardo García-Del-Rey, Aya Goldshtein, Anne E Goodenough, Andrew G Gosler, Iga Góźdź, Arnaud Grégoire, Lars Gustafsson, Ian R Hartley, Philipp Heeb, Shelley A Hinsley, Paul Isenmann, Staffan Jacob, Antero Järvinen, Rimvydas Juškaitis, Erkki Korpimäki, Indrikis Krams, Toni Laaksonen, Bernard Leclercq, Esa Lehikoinen, Olli Loukola, Arne Lundberg, Mark C Mainwaring, Raivo Mänd, Bruno Massa, Tomasz D Mazgajski, Santiago Merino, Cezary Mitrus, Mikko Mönkkönen, Judith Morales-Fernaz, Xavier Morin, Ruedi G Nager, Jan-Åke Nilsson, Sven G Nilsson, Ana C Norte, Markku Orell, Philippe Perret, Carla S Pimentel, Rianne Pinxten, Ilze Priedniece, Marie-Claude Quidoz, Vladimir Remeš, Heinz Richner, Hugo Robles, Seppo Rytkönen, Juan Carlos Senar, Janne T Seppänen, Luís P da Silva, Tore Slagsvold, Tapio Solonen, Alberto Sorace, Martyn J Stenning, János Török, Piotr Tryjanowski, Arie J van Noordwijk, Mikael von Numers, Wiesław Walankiewicz, Marcel M Lambrechts

**Affiliations:** 1 Laboratoire Ecologie, Systematique et Evolution, UMR 8079 CNRS-Université Paris-Sud XI-AgroParisTechBatiment 362 Université Paris-Sud XI, Orsay Cedex, F-91405, France; 2 Evolutionary Ecology Group, Department of Biology, University of AntwerpCampus CGB, Antwerp, B-2020, Belgium; 3 Russian Academy of Sciences, Karelian Research Centre, Instition of BiologyPetrozavodsk, 185610, Russia; 4 Department of Experimental Zoology & Evolutionary Biology, University of ŁodźBanacha 12/16, Łodź, 90-237, Poland; 5 Terrestrial Vertebrates Research Unit “Cavanilles”, Institute of Biodiversity and Evolutionary Biology, University of ValenciaC/Catedrático José Beltran 2, Paterna, E-46980, Spain; 6 Laboratoire Ecologie-Evolution, UMR 7625, Equipe Ecophysiologie Evolutive - Evolutionary Ecophysiology Research Group, Université Pierre et Marie Curie – UPMC7 quai Saint Bernard, case 237, Paris Cedex 05, F-75252, France; 7 Centre d’Ecologie Fonctionnelle et Evolutive, CEFE UMR 5175Campus CNRS, 1919 route de Mende, Montpellier Cedex 5, F-34293, France; 8 Research Laboratory “Ecology of Terrestrial and Aquatic Systems”, University Badji MokhtarAnnaba, Algeria; 9 INRA, UR 1115, Plantes et Systèmes de culture HorticolesAvignon, F-84000, France; 10 Àrea de Biodiversitat, Grup de Biologia de la Conservació, Centre Tecnològic Forestal de CatalunyaCarretera de St. Llorenç de Morunys, km. 2, Solsona, E-25280, Spain; 11 Strada Bine1424, Acquanegra sul Chiese (MM), I-46011, Italy; 12 Centre d’Ecologie Fonctionnelle et Evolutive, CEFE UMR 5175Campus CNRS, 1919 route de Mende, Montpellier Cedex 5 F-34293, France; 13 University of HaifaHaifa, Israel; 14 Society for the Protection of Nature, University of LausanneLausanne, Switzerland; 15 Institution of Environment Science, Jagiellonian UniversityKrakow, Poland; 16 Stazione Inanellamento c/o Dipartimento SEN-FIMIZO, Università di PalermoPalermo, Italy; 17 Department of Zoology, Siedlce University of Natural Sciences and HumanitiesPrusa 12, Siedlce, PL-08-110, Poland; 18 Centre d’Ecologie Fonctionnelle et Evolutive, CEFE UMR 5175Campus CNRS, 1919 route de Mende, Montpellier Cedex 5 F-34293, France; 19 Univ Lyon 1, Department of Biometry & Evolutionary Biology, CNRS UMR 5558Villeurbanne, F-69622, France; 20 Centre d’Ecologie Fonctionnelle et Evolutive, CEFE UMR 5175Campus CNRS, 1919 route de Mende, Montpellier Cedex 5 F-34293, France; 21 Museum and Institute of Zoology, Polish Academy of SciencesWilcza 64, Warsaw, PL-00-679, Poland; 22 Campus Drie Eiken, Department of Biology (Ethology)Building C, Antwerp (Wilrijk), B-2610, Belgium; 23 Section of Ecology, Department of Biology, University of TurkuTurku, FI-20014, Finland; 24 Université de Bourgogne, UMR CNRS 5561, BioGéoSciences6 Boulevard Gabriel, Dijon, F-21000, France; 25 School of Bioscience, Cardiff UniversityCardiff, CF10 3AX, UK; 26 Department of Biology, University of OuluOulu, FIN-90014, Finland; 27 Departamento de Ecología, Facultad de Biología, Universidad de La LagunaLa Laguna, E-38260, Spain; 28 Department of Natural and Social Sciences, University of GloucestershireGloucestershire, GL50 4AZ, U.K; 29 Department of Zoology, Edward Grey Institute of Field Ornithology & Institute of Human SciencesSouth Parks Road, Oxford, OX1 3PS, U.K; 30 Museum and Institute of Zoology, Polish Academy of SciencesWilcza 64, Warszawa, PL-00-679, Poland; 31 Centre d’Ecologie Fonctionnelle et Evolutive, CEFE UMR 5175Campus CNRS, et Université de Montpellier II, 1919 route de Mende, Montpellier Cedex 5, F-34293, France; 32 Department of Animal Ecology, Evolutionary Biology Centre, Uppsala UniversityUppsala, SE-75236, Sweden; 33 Lancaster Environment Centre, Lancaster UniversityLancaster, LA1 4YQ, U.K; 34 Laboratoire Évolution & Diversité Biologique, UPS Toulouse III, Bât 4R1, salle 122118 route de Narbonne, Toulouse, F-31062, France; 35 CEH WallingfordMaclean Building, Crowmarsh Gifford, Oxfordshire, OX10 8BB, U.K; 36 Centre d’Ecologie Fonctionnelle et Evolutive, CEFE UMR 5175Campus CNRS, 1919 route de Mende, Montpellier Cedex 5 F-34293, France; 37 Laboratoire Évolution & Diversité Biologique, UPS Toulouse III, Bât 4R1, salle 122118 route de Narbonne, Toulouse F-31062, France; 38 University of HelsinkiKilpisjarvi Biological Station, P.O.Box 17, Helsinki, FIN-00014, Finland; 39 Institute of Ecology of Nature Research CentreAkademijos 2, Vilnius, LT-08412, Lithuania; 40 Section of Ecology, Department of Biology, University of TurkuTurku FI-20014, Finland; 41 Institute of Ecology & Earth Sciences, University TartuTartu, EE-51014, Estonia; 42 Section of Ecology, Department of Biology, University of TurkuTurku FI-20014, Finland; 43 Crx. St. Pierre6 rue Morcueil, Fleurey Sur Ouche, F-21410, France; 44 Section of Ecology, Department of Biology, University of TurkuTurku FI-20014, Finland; 45 Department of Biology, University of OuluOulu, FI-90014, Finland; 46 Department of Ecology & Evolution, Uppsala UniversityUppsala, S-75236, Sweden; 47 Lancaster Environment Centre, Lancaster UniversityLancaster LA1 4YQ, U.K.; 48 Department of Zoology, Institute of Ecology and Earth Sciences, University of Tartu46 Vanemuise Str., Tartu, EE-51014, Estonia; 49 Stazione Inanellamento c/o Dipartimento SEN-FIMIZO, Università di PalermoPalermo, Italy; 50 Museum and Institute of Zoology, Polish Academy of Sciences, Wilcza 64Warszawa PL-00-679, Poland; 51 Departamento de Ecología Evolutiva Museo Nacional de Ciencias Naturales, Agencia Estatal Consejo Superior de Investigaciones Científicas, CSICC/José Gutiérrez Abascal 2, Madrid, E-28002, Spain; 52 Department of Zoology, Rzeszów UniversityZelwerowicza 4, Rzeszów, PL-35-601, Poland; 53 Department of Biological and Environmental Sciences, University of JyväskyläPOB 35, Jyväskylä, FIN-40014, Finland; 54 Centre d’Ecologie Fonctionelle & Evolutive, CNRS1919 Route de Mende, Montpellier, France; 55 Ecología Evolutiva, Museo Nacional de Ciencias Naturales (CSIC)José Gutiérrez Abascal 2, Madrid, E-28006, Spain; 56 Centre d’Ecologie Fonctionnelle et Evolutive, Campus CNRS1919 route de Mende, Montpellier Cedex 5, F-34293, France; 57 Institute of Biodiversity, Animal Health & Comparative Medicine, University of GlasgowGraham Kerr Building, Glasgow, G12 8QQ, U.K; 58 Ecology Building, Animal Ecology, Lund UniversityLund, SE-223 62, Sweden; 59 Department of Biology, Biodiversity, Lund UniversityEcology Building, Lund, SE-223 62, Sweden; 60 Department of Life Sciences, Institute of Marine Research, University of CoimbraApartado 3046, Coimbra, PT-3001-401, Portugal; 61 Department of Biology, University of OuluP.O. Box 3000, Oulu, FIN-90014, Finland; 62 Centre d’Ecologie Fonctionnelle et Evolutive, CEFE UMR 5175Campus CNRS, 1919 route de Mende, Montpellier Cedex 5 F-34293, France; 63 Centro de Estudos Florestais, Instituto Superior de Agronomia, University of LisbonLisbon, 1349-017, Portugal; 64 Campus Drie Eiken, Department of Biology (Ethology), Building CAntwerp (Wilrijk) B-2610, Belgium; 65 Latvian Fund for NatureDzirnavu Street 73-2, Riga, LV-1011, Latvia; 66 Centre d’Ecologie Fonctionnelle et Evolutive, CEFE UMR 5175Campus CNRS, 1919 route de Mende, Montpellier Cedex 5 F-34293, France; 67 Laboratory of Ornithology, Department of Zoology, Palacky UniversityOlomouc, CZ-77146, Czech Republic; 68 Institute of Ecology & Evolution (IEE), University of BernBern, CH-3012, Switzerland; 69 Falculty of Sciences, Evolutionary Ecology Group (GIBE), University of A CoruñaCampus Zapateira, A Coruña, E-15008, Spain; 70 Evolutionary Ecology Group (EVECO), Department of Biology, University of AntwerpMiddelheimcampus, Groenenborgerlaan 171, Antwerp, B-2020, Belgium; 71 Department of Biology, University of OuluP. O. Box 3000, Oulu, FIN-90014, Finland; 72 Unidad Asociada CSIC de Ecología Evolutiva y de la Conducta, Nat-Museu de Ciències Naturals de BarcelonaBarcelona, Spain; 73 Department of Biological and Environmental Science, University of JyväskyläJyväskylä, Finland; 74 Department Life Science, IMAR CMA, University CoimbraCoimbra, PT-3004517, Portugal; 75 Department of Biology, University of OsloOslo, Norway; 76 Luontotutkimus Solonen OyNeitsytsaarentie 7b B 147, Helsinki, FIN-00960, Finland; 77 SROPUVia R. Crippa 60, Rome, Italy; 78 School of Life Sciences, University of SussexBrighton, Sussex, BN1 9QG, U.K; 79 Behavioral Ecology Group, Department of Systematic Zoology & Ecology, Eötvös Lorand UniversityBudapest, H-1117, Hungary; 80 Institute of Zoology, Poznan University of Life SciencesWojska Polskiego 71 C, Poznań, PL-60-625, Poland; 81 Netherlands Institute of Ecology (NIOO-KNAW)Doevendaalsesteg, 10, Wageningen, NL-6708 BP, the Netherlands; 82 Environmental and Marine Biology, Åbo Akademi UniversityArtillerigatan 6, Biocity, Åbo, FI-20520, Finland; 83 Department of Zoology, Siedlce University of Natural Sciences and Humanities, Prusa 12Siedlce PL-08-110, Poland; 84 Centre d’Ecologie Fonctionnelle et Evolutive, CEFE UMR 5175Campus CNRS, 1919 route de Mende, Montpellier Cedex 5 F-34293, France

**Keywords:** Hole nesting, natural holes, nest boxes, reaction norm

## Abstract

Nests are structures built to support and protect eggs and/or offspring from predators, parasites, and adverse weather conditions. Nests are mainly constructed prior to egg laying, meaning that parent birds must make decisions about nest site choice and nest building behavior before the start of egg-laying. Parent birds should be selected to choose nest sites and to build optimally sized nests, yet our current understanding of clutch size-nest size relationships is limited to small-scale studies performed over short time periods. Here, we quantified the relationship between clutch size and nest size, using an exhaustive database of 116 slope estimates based on 17,472 nests of 21 species of hole and non-hole-nesting birds. There was a significant, positive relationship between clutch size and the base area of the nest box or the nest, and this relationship did not differ significantly between open nesting and hole-nesting species. The slope of the relationship showed significant intraspecific and interspecific heterogeneity among four species of secondary hole-nesting species, but also among all 116 slope estimates. The estimated relationship between clutch size and nest box base area in study sites with more than a single size of nest box was not significantly different from the relationship using studies with only a single size of nest box. The slope of the relationship between clutch size and nest base area in different species of birds was significantly negatively related to minimum base area, and less so to maximum base area in a given study. These findings are consistent with the hypothesis that bird species have a general reaction norm reflecting the relationship between nest size and clutch size. Further, they suggest that scientists may influence the clutch size decisions of hole-nesting birds through the provisioning of nest boxes of varying sizes.

## Introduction

Numerous organisms, including insects, spiders, crustaceans, fish, amphibians, reptiles, birds, and mammals, all construct nests that are used for containing their eggs and/or offspring for a considerable amount of time (Collias and Collias 1984; Taylor et al., 1998; Hansell 2000, 2007). Nest size depends on nest site, and choice of a nest hole as a breeding site may constrain the size of nests. Hence, nest size in hole nesters may depend on the size of the cavity or the nest site. Nests are often larger than the builder(s) themselves and sometimes considerably so, as shown by ants, termites, and some birds such as raptors (Collias and Collias 1984; Hansell 2000, 2007). Nest building is an energetically costly and time-consuming activity that is presumably traded-off against participation in other activities (Collias and Collias 1984; Hansell 2000, 2007; Mainwaring and Hartley 2013).

The parental expenditure on nest building can appear to be paradoxical as nests are often much larger than is required for successful reproduction (Soler et al. 1998a,b; and references below). This poses the question as to why animals build nests that are larger than is necessary for raising the offspring (Mainwaring and Hartley 2013).

There are six main, nonmutually exclusive, reasons why large nests may be advantageous. First, the maintenance of a specific nest temperature and humidity (Mertens 1977a,b; Erbeling-Denk and Trillmich 1990; Mainwaring et al. 2012). Optimal nest size may provide a microenvironment that is not too cooled or too over-heated for growth and development of temperature regulation (Mertens 1977a,b; Erbeling-Denk and Trillmich 1990; Mainwaring et al. 2012). Larger and better insulated nests built at higher latitudes (Schaefer 1980; Møller 1984; Crossman et al. 2011; Mainwaring et al. 2012), higher altitudes (Kern and Van Riper 1984), and early during the season when temperatures are cooler at temperate latitudes (Møller 1984; Mainwaring and Hartley 2008) are consistent with thermal benefits of large nests at extreme temperatures. Second, specific types of nest material may provide protection of eggs and nestlings against bacteria and parasites (Wimberger 1984; Mennerat et al. 2009; Peralta-Sánchez et al. 2010). Third, an optimal nest size will prevent excessive fouling of nests and the associated fitness costs of nestling death by allowing parents to keep the nest clean, as demonstrated experimentally for starlings *Sturnus vulgaris* by Erbeling-Denk and Trillmich (1990). Fourth, sexual selection (Tortosa and Redondo 1992; Soler et al. 1998a,b; Møller 2006; Broggi and Senar 2009; Sanz and García-Navas 2011; Tomás et al. 2013). Soler et al. (1998a) reported for European passerines the relationship between nest size and the number of parent birds involved in nest building, and found that nest size almost doubled when both male and female build nests as compared to nests built by females alone. This was interpreted to have arisen because both parents signal their phenotypic quality via their investment in nest building, despite the fact that clutch size did not differ between species in which only females build nests and species in which both sexes build. This interpretation is supported by intraspecific correlational and experimental studies showing that larger nests are favored because of sexual selection (Soler et al. 1998b, 1999, 2001; de Neve et al. 2004; Tomás et al. 2013). Fifth, structural support may prevent eggs and nestlings from being lost. Large nests may benefit parent birds through improved insulation and the reduced heat loss from eggs and nestlings. Sixth, large nests may reduce crowding of the offspring and prevent them from falling out of the nest (Slagsvold 1982, 1989; Heenan and Seymour 2011). Thus, reproductive success is positively correlated with nest size (Alabrudzinska et al. 2003; Álvarez and Barba 2008), raising the question as to why nest size does not continue to increase.

Nest sizes are restricted by five main selective forces. First, the risk of nest predation and parasitism selects for smaller nest sizes by disproportionately affecting larger nests (Møller 1990; Eeva et al. 1994; Soler et al. 1999; Antonov 2004). Second, building larger nests means more time spent collecting nesting materials, which are often found on the ground, increasing the risk of predation on nest-building adults (Slagsvold and Dale 1996). Third, large and consequently warm and humid nests do not only benefit offspring, but also pathogens and parasites that inhabit nests, and bacteria multiply at higher rates in warm and humid nest environments compared to ambient temperatures, with potential costs to their avian inhabitants (Stolp 1988**)**. Some nest-building birds have evolved counter-adaptations against exploitation by parasites by using nest material with antimicrobial properties such as feathers and plants with secondary defense substances (Clark and Mason 1985; Gwinner 1997; Mennerat et al. 2009; Møller et al. 2013) or uropygial secretions that eliminate bacteria from eggs (Soler et al. 2012). Fourth, nest size may be limited by individual quality and the construction of larger nests when males participate in nest building may have a signaling function, reflecting the working ability of males for finding specific or scarce nest materials (Møller 2006; Broggi and Senar 2009; Sanz and García-Navas 2011). Fifth, nest size may increase the risk of brood parasitism (Soler et al. 1995, 1999), or decrease the probability of rejection of host offspring by brood parasites (Anderson et al. 2009; Grim et al. 2009). These effects seem minor given that most hole nesters are only rarely parasitized by parasitic cuckoos (Moksnes et al. 1991; Moksnes and Røskaft 1995; Davies 2000).

Despite the many and varied factors selecting for or against larger nests, a major factor is likely to be clutch size. Clutch size is often larger when nests are larger (Löhrl 1973, 1980; Karlsson and Nilsson 1977; van Balen 1984; Gustafsson & Nilsson, 1985; Korpimäki 1985; Rendell and Robertson 1993; Wiebe and Swift 2001). This is surprising because nests are generally made well before the first eggs are laid, although parent birds sometimes make adjustments to nests during incubation and even as late as the nestling period (Møller 1987), posing a potential problem for parents at the time of nest building due to uncertainty about final clutch size. This relationship may arise from both clutch size and nest size being correlated negatively with laying date. This cannot be the entire explanation for experimental studies such as those by Löhrl (1973, 1980), Erbeling-Denk and Trillmich (1990), Rendell and Robertson (1993), and Soler et al. (2001), who documented a change in clutch size when nest box size was manipulated after the start of egg laying. These experimental studies showed effects of nest size on clutch size independent of potentially confounding variables. On average, a larger nest after laying is required for raising more offspring. There may be a correlation between the choice of nest site by females and the subsequent clutch size, females building larger nests because such females lay larger clutches, or females laying larger clutches may build larger nests. Several experiments have tried to separate the effects of the size of the nest cavity and the effects of female phenotype on subsequent clutch size by changing nest size through exchanging nest boxes of different sizes after the female had started laying. Exchange experiments performed during egg laying in the great tit resulted in an increase in clutch size when box size increased, but a decrease when a smaller box was provided (Löhrl 1973, 1980; Erbeling-Denk and Trillmich 1990; Rendell and Robertson 1993).

The objective of this study was to investigate the plasticity of clutch size in response to nest size in both hole and open nesting birds. We included all species for which information on nest size and clutch size was available in these analyses because females faced the problem of adjusting clutch size to nest size before hole nesting evolved, and because hole nesting is a derived character in birds (Lack 1968). Therefore, adjustment of clutch size to nest size is likely to have evolved before hole nesting. First, using data in classical experiments on great tits by Löhrl (1973, 1980), we investigated the effects of nest box size on clutch size, when great tits had access to differently sized nest boxes, and when the size of nest boxes was manipulated after laying had started. The latter design prevented differential access of specific females to boxes of a particular size. Second, we examined the plasticity of clutch size to nest size in hole nesters where nest box sizes were varied experimentally within a population. Third, we analyzed the relationship between clutch size and nest size using studies of four secondary hole-nesting species with both single or multiple nest box sizes. We predicted that if hole-nesting birds use a single type of plasticity as a clutch size response to cavity size, then we should find a homogeneous slope linking clutch size to cavity size. Fourth, if different species of hole-nesters respond differently to cavity size, for example, because populations in different environments encounter different problems in terms of hyperthermia, we should expect heterogeneous relationships between clutch size and cavity size. Finally, we predicted that the slope of the relationship between clutch size and nest size depended on species, latitude and longitude if climatic conditions were underlying variation in slope among studies. We emphasize that the results presented by Møller et al. (2014) only constitute one out of 11 analyses reported here. These previous analyses of 365 populations with more than 79,000 clutches showed that clutch size in the great tit *Parus major*, but not in three other species of hole nesters increased with nest box size, and that nest box size and quality varied nonrandomly among populations (Møller et al. 2014). Here, we broaden this approach to a larger number of species, including open nesting species, by exploring the causality of this relationship and analyzing whether plasticity of clutch size in relation to nest size vary between species and/or regions when correcting for macrogeographic and habitat patterns. Open nesting species differ from hole nesters by being less constrained by nest cavity size as hole nesters. This approach will give insight about the factors governing the relationship between clutch and nest size.

## Materials and Methods

### Data sets

This study was based on three different data sets: (1) Löhrl’s (1973, 1980) data on great tits; (2) a data base on clutch size and nest box size in four secondary hole-nesting birds; and (3) a exhaustive database on the relationship between clutch size and nest size in 21 species of birds for which data on clutch size and nest size were available (Table S1). We defined nest size in hole-nesters as the two-dimensional area of the nest box or nest hole in case the study was based on natural cavities (i.e., nest base area). Similarly, we defined nest size for open nesting species as the horizontal maximum area of the nest including nest cup and nest rim (i.e., nest base area). Nest size refers to this definition in the remainder of the article. We used this measure of nest size because it was available for almost all studies. We could not use total volume of the nest or nest weight as a measure of nest size because only a small number of studies had recorded and/or published such data. Use of our definition of nest size relies on the assumption that nest volume and/or nest weight is positively correlated with nest size as defined here. We were able to explicitly test this assumption for nest size (calculated as 4/3 × π × a^2^ × b, where a is the radius and b is the height) in five studies of four species, two hole nesters and two open nesters: blue tits (M. Lambrechts et al., unpubl. data: *F*_1,52_ = 176.68, *r*^2^ = 0.77, *P* < 0.0001, slope (SE) = 1.484 (0.112)), great tits (M. Lambrechts et al., unpubl. data: *F*_1,212_ = 634.07, *r*^2^ = 0.75, *P* < 0.0001, slope (SE) = 1.280 (0.050)), and great tits (Slagsvold and Amundsen 1992: *F*_1,27_ = 207.07, *r*^2^ = 0.99, *P* < 0.0001, slope (SE) = 1.197 (0.083)), the open nesting barn swallows *Hirundo rustica* (A. P. Møller, unpubl. data: *F*_1,1265_ = 866.17, *r*^2^ = 0.40, *P* < 0.0001, slope (SE) = 1.202 (0.041)) and rufous bush warblers *Cercotrichas galactotes* (M. Martín-Vivaldi, unpubl. data: *F*_1,48_ = 24.28, *r*^2^ = 0.34, *P* < 0.0001, slope (SE) = 0.937 (0.190)) using log_10_-transformed data.

We extracted data from Löhrl’s (1973, 1980) pioneering studies of great tit clutch size, choice of nest box size and experimental manipulation of nest box size after start of laying. We used these data to estimate the relationship between nest size and clutch size, and in the nest box exchange experiment the relationship between clutch size and the change in nest box size.

We made an exhaustive attempt to obtain information on clutch size, nest size and ecological variables from all studies of four common species of secondary hole nesters in Europe and North Africa, as described in detail elsewhere (Møller et al. 2014). Briefly, we attempted to obtain data on first clutches, or early clutches known to be initiated less than 30 days after the first egg was laid in a given year in a local study plot (cf. Nager and van Noordwijk 1995). In total, we obtained information on 155 study populations of great tits, 121 of blue tits *Cyanistes caeruleus*, 24 of pied flycatchers *Ficedula hypoleuca*, and 65 of collared flycatchers *Ficedula albicollis* for a total of 1479 study years for great tits, 1122 for blue tits, 288 for pied flycatchers, and 592 for collared flycatchers, or in total 79,610 clutches (Møller et al. 2014).

For all studies, we also quantified latitude (°N) and longitude (°E), main habitat type (deciduous, coniferous, evergreen, or mixed), urbanization (urbanized, or natural/semi-natural habitat), altitude at the center of a study plot, mean study year, nest floor surface as the internal nest base area (in cm²), and the material used to construct nest boxes (a binary variable classified as either wood or concrete). We defined minimum and maximum size of boxes for all study sites that used two or in one case three nest box sizes by assigning the smallest box size as the minimum size and the largest box size as the maximum size. Further details of how these variables were obtained and quantified can be found in Lambrechts et al. (2010) and Møller et al. (2014).

We made an exhaustive literature search for papers dealing with clutch size and nest size in birds using Web of Science (clutch size, nest size, and birds were used as keywords) combined with a search for other publications in the reference lists of these papers (Lambrechts et al. 2010; Møller et al. 2014). If there was missing information on clutch size or nest size in these publications, we attempted to retrieve this information by contacting the authors. The extensive data set on clutch size and nest size in barn swallows was collected by APM by measuring inner and outer diameter and inner depth of all nests in a Danish study site during 1988–2012 (see Møller (2006) for further details).

### Ecological characteristics

All studies that allowed an estimation of slope of the relationship between clutch size and base area of the nest were cross-classified with respect to (1) hole nesting, (2) box or natural hole, (3) nest building, (4) body mass and (5) minimum, and (6) maximum size of nests used in a given study to test for an association between the slope of the relationship between clutch size and nest size for each year for all study populations. Species were classified as (1) hole nesters (yes – breeding in cavities, no – not breeding in cavities), (2) nest builders (yes – building or excavating the nest or the cavity, no – not building or excavating the nest or the cavity), as reported in Cramp and Perrins (1977–1994), Dunning (1993) and Poole (2005), and (3) nest box populations (yes, no). All studies with slope estimates of the relationship between clutch size and nest size as defined above and the ecological data are listed in the Table S1.

### Statistical analyses

This study presents the results of 11 statistical analyses of the relationship between clutch size and nest size within and among species of birds (we have found no comparable data on any other taxa). This study differs from that by Møller et al. (2014) by analyzing an exhaustive compilation of data on both hole and open nesting species and a wider range of factors hypothesized to affect the relationship between clutch size and nest size.

We re-analyzed Löhrl’s (1973, 1980) data to allow distinction between the effects of original and subsequent nest box size on clutch size following exchange of nest boxes during the laying period. We did so by analyzing clutch size (the response variable) in relation to original and experimental nest box size.

We estimated slopes of the relationship between clutch size and nest base area. These analyses were based on all studies with at least two different sizes of nest boxes (all but one study had two box sizes only), where the analyses were weighted by sample size for the different categories of nest box sizes. Similarly for open nesting species, we estimated slopes by relying on clutch size and base area of nests.

We have previously investigated the relationships between the average size of first clutches and internal base area (cm^2^) of artificial cavities after controlling for other factors influencing average clutch size (Møller et al. 2014). We used mixed models (GLMM) including (1) plot ID as a random effect to account for differences in sample sizes among plots, and (2) species (great tit, blue tit, pied flycatcher, collared flycatcher) as a random factor that accounted for the difference in level of sampling among species and the following variables as factors: (3) latitude (32–65°N), (4) longitude (9°W–35°E), (5) dominant vegetation (deciduous, coniferous, evergreen, mixed), (6) degree of urbanization (urban, rural), (7) altitude (0–1650 m a. s. l.), (8) cavity type (artificial box, tree cavity), (9) material (cavity wall made of wood, cavity wall made of material other than wood, mainly wood-concrete), (10) year (average study year for studies that lasted >1 year), and (11) study period (number of study years). We log_10_-transformed altitude to eliminate the skewed distribution adding a constant of 192 m to avoid inclusion of negative values recorded in a few study sites below sea level. We weighted the analyses by sample size to account for the fact that estimated clutch sizes based on large sample sizes will be closer to the actual population mean than those based on small sample sizes.

In the current study, we tested for a difference in slope among 116 populations of hole-nesting and open-nesting bird species using mixed models with slopes weighted by sample size as the response variable and species as a fixed factor (21 species), and latitude and longitude as covariates. Because transformations did not result in normal distributions, we used ranked slopes as a response variable in this analysis. In addition, we used Welch ANOVA for unequal variances weighted by sample size to test for differences in means and variances among species, again relying on ranked slopes. Finally, we also included into our least squares models the minimum and maximum size of nests/boxes used in each study to test for their effects on slope estimates. The justification for this approach was that minimum size would reflect the importance of hyperthermia created in the crammed conditions of small nests, while maximum nest size would reflect problems of hypothermia (Mertens 1977a,b).

When quantifying the effects of base area of nests on clutch size we estimated the change in clutch size for an increase in one standard deviation unit in base area using the linear regression coefficient. In all 11 statistical tests presented in Table 1 we provide the mean and the standard error of the parameter estimates. These were subsequently used to estimate the 95% confidence intervals (CI) for the estimated slopes. If 95% CI of two or more samples did not overlap, they were considered to be significantly different. All statistical tests were made with JMP (SAS 2012).

**Table 1:** Relationship between clutch size and nest base area in different studies and their 95% confidence intervals (CI)

Relationship	Species	*F*	df	*P*	Estimate (SE)	95% CI
A. Clutch size and base area in choice test (Löhrl 1973)	*Parus major*	28.61	1, 49	<0.0001	0.0088 (0.0016)	0.0056, 0.0120
B. Clutch size and base area when box is exchanged during laying (Löhrl 1980)	*Parus major*	6.48	1, 18	0.020	0.0264 (0.0104)	0.0045, 0.0483
C. Clutch size and change in base area when box is exchanged during laying (Löhrl 1980)	*Parus major*	6.48	1, 18	0.020	0.0132 (0.0052)	0.0033, 0.0241
D. Clutch size and base area in studies based on two or more nest box sizes	*Parus major, Cyanistes caeruleus, Ficedula hypoleuca, Ficedula albicollis*	6.50	1, 68	<0.0001	0.0036 (0.0014)	0.0009, 0.0063
E. Clutch size and base area in all studies of four secondary hole nesters	*Parus major, Cyanistes caeruleus, Ficedula hypoleuca, Ficedula albicollis*	3.37	1, 3371	0.067	−0.0211 (0.0115)	−0.044, 0.0014
F. Clutch size and base area in great tit	*Parus major*	24.31	1, 1434	<0.0001	0.0071 (0.0014)	0.0044, 0.0098
G. Clutch size and base area in blue tit	*Cyanistes caeruleus*	2.11	1, 382.2	0.15	−0.0038 (0.0026)	−0.0089, 0.0013
H. Clutch size and base area in pied flycatcher	*Ficedula hypoleuca*	0.65	1, 22.41	0.43	−0.0016 (0.0020)	−0.0055, 0.0023
I. Clutch size and base area in collared flycatcher	*Ficedula albicollis*	0.00	1, 32.29	0.99	0.000013 (0.0020)	−0.0039, 0.0039
J. Clutch size and base area in all open-nesting species	Open-nesters	9.90	1, 25	0.0037	0.0033 (0.0010)	0.0013, 0.0054
K. Clutch size and base area in all species	All species	12.51	1, 114	0.0006	0.0036 (0.0010)	0.0056, 0.0063

## Results

### Clutch size and nest size in Löhrl’s studies of great tits

First, we investigated whether the relationship between clutch size and nest box base area was significant when great tits could choose between boxes that had base area of either 64 or 314 cm^2^, using data reported by Löhrl (1973). This relationship was indeed significantly positive (Table 1A, slope (SE) = 0.0088 eggs cm^−2^ (0.0016)).

Second, Löhrl (1980) provided experimental evidence for great tits adjusting their clutch size directly to the base area of nest boxes by exchanging the nest boxes during the laying period. This justifies that the analyses with clutch size as a response variable and nest base area as a predictor variable were made without inclusion of additional variables. The relationship between clutch size and box base area was statistically significant (Table 1B, slope (SE) = 0.0264 (0.0104)). This relationship and that reported when great tits were allowed to choose box size were not significantly different, as shown by their overlapping 95% CI (Table 1). These data also allowed a test of whether the relationship between clutch size and change in box base area between the original and the new box was statistically significant. That was indeed the case. The slope was half that of the previous estimate (Table 1C), although the 95% CI of the parameter estimate overlapped with the previous two estimates (Table 1A, B). Mean nest base area in the second dataset for great tits weighted by sample size across all 3447 samples was 118 cm^2^, range 36–400 cm^2^, SD = 123 cm^2^. This implies that an increase by one SD in nest base area was associated with an average increase in clutch size of the great tit by 0.0088 eggs cm^−2^ × 123 cm^2^ = 1.08 eggs to 0.0264 eggs cm^−2^ × 123 cm^2^ = 3.25 eggs for these two extreme estimates of the relationship.

### Clutch size and nest size across species of birds

We obtained an estimate of the clutch size – nest base area relationship in population studies of all four species of hole-nesting birds (great tits, blue tits, pied flycatchers, and collared flycatchers) with more than one box size since a slope estimate requires at least two box sizes within a study site. The mean slope weighted by sample size was 0.0036 (SE = 0.0014), *N* = 69 populations and four species (Table 1D). This mean slope estimate was not significantly different from those reported by Löhrl (1973, 1980), as shown by the overlapping 95% CI (Table 1).

We then estimated the relationship between clutch size and base area in populations with one or more box sizes for the four secondary hole nesters considered above. This relationship was statistically significant for all species combined (Table 1E). It was also statistically significantly positive for great tit separately (Table 1F), but not for blue tit (Table 1G), collared flycatcher (Table 1H) or pied flycatcher (Table 1I). The interaction between species and nest floor area was highly significant (*F*_3,3436_ = 115.12, *P* < 0.0001), implying that clutch size was related to nest floor area in a species-specific manner. A Welch analysis of variance for unequal variances with ranked slope as the response variable, species as a fixed factor and sample size as a weight revealed a significant effect of means of species (*F*_7,3660.6_ = 6.23, *P* < 0.0001), and a Levene’s test showed a significant difference in variance among species (*F*_7,95_ = 5.06, *P* < 0.0001). Thus, a general positive relationship between clutch size and nest size differed significantly among species, but the variances within species also differed significantly. The overall slope estimate was not significantly different from those reported by Löhrl (1973, 1980) for great tits, as shown by the overlapping 95% CI (Table 1).

Open-nesting species also showed an overall positive relationship between clutch size and base area (Table 1J) that overlapped with all the slope estimates reported in Table 1 except for the estimate for clutch size and nest base area in Löhrl’s (1973) choice test (Table 1A). We tested whether the relationship between clutch size and base area was statistically significant across all species by inclusion of hole-nesters and open-nesters. The overall slope estimate was statistically significant (Table 1K). It overlapped with most of the slope estimates reported in Table 1 suggesting that even inclusion of open-nesters in an overall estimate across species did not change the conclusion that there was a general positive relationship between clutch size and base area in birds.

**Figure 1:**
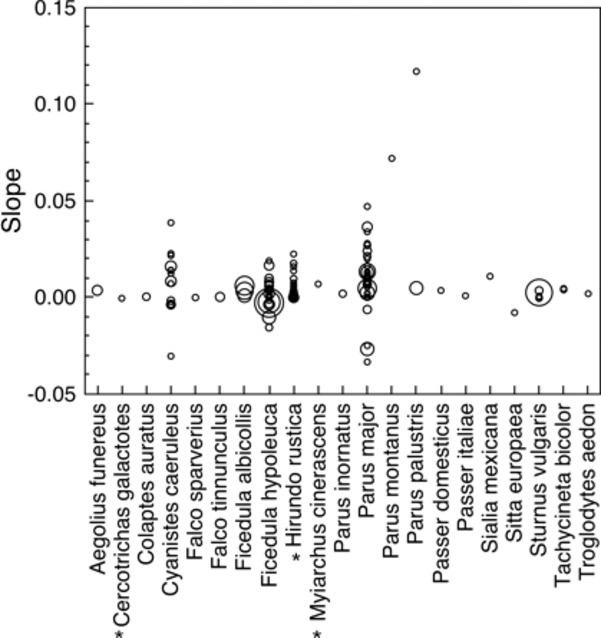
Slopes of the relationship between nest base area and clutch size in different species of birds in each study population. The three open-nesting species are shown with an *. Note that symbol size is related to the number of nests.

**Figure 2:**
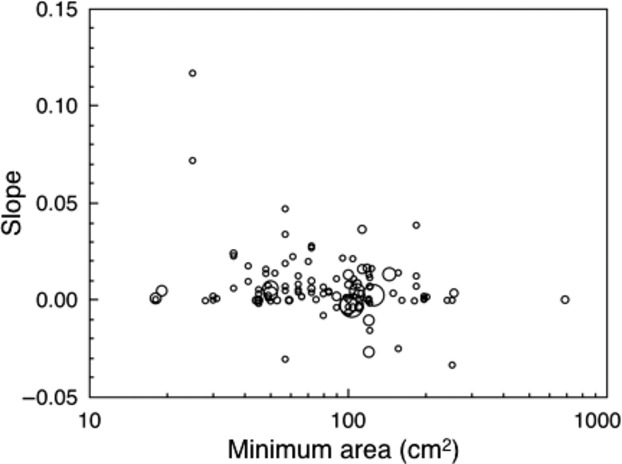
Slopes of the relationship between nest base area and clutch size in relation to the minimum area (cm^2^) of nest box bases in different populations and species of birds. Note that symbol size is related to the number of nests.

We tested whether there was a species-specific relationship between clutch size and nest base area in the data set based on study sites where more than one box size was used and more than one nest size was recorded (Fig. 1). This test was based on all studies for which we had information on the regression coefficient between clutch size and nest base area. Based on these 116 datasets, a Welch analysis of variance for unequal variances with ranked slope as the response variable, species as a fixed factor and sample size as a weight revealed a significant effect of means of species (Fig. 2; *F*_7,3660.6_ = 6.23, *P* < 0.0001), and a Levene’s test showed a significant ’difference in variance among species (*F*_7,95_ = 5.06, *P* < 0.0001). This shows a general positive relationship that differed significantly among species, and the variances within species also differed significantly.

Because ambient temperatures during the breeding season are predictably warmer at lower latitudes, we expected a stronger relationship between clutch size and nest base area in southern populations than in northern populations. However, adding latitude (*F*_1,93_ = 1.43, *P* = 0.23) or longitude (*F*_1,93_ = 1.02, *P* = 0.31) or the squared values to account for nonlinear effects (latitude squared: *F*_1,92_ = 0.01, *P* = 0.90; longitude squared: *F*_1,92_ = 0.04, *P* = 0.85) to the model for the 21 species did not show significant effects. A least squares model with species as a fixed effect showed a significant negative association between slope of the relationship between clutch size and nest size and minimum size (Table 2; Fig. 2) and maximum size of boxes in a given study (Table 2). The effect of species only accounted for 2.7% of the variance and hence was negligible. The effect of minimum size was almost twice as large as the effect of maximum size. All variance inflation factors were smaller than 5 and hence did not pose a problem of collinearity (McClave and Sincich 2003),

**Table 2:** Least squares model of the relationship between ranked slope relating clutch size to minimum and maximum base area and species (fixed effect). The model had the statistics *F* = 2.68, df = 22, 93, *r*^2^ = 0.24*, P* = 0.0005

Variable	Sum of squares	df	*F*	*P*	Estimate	Error
Intercept			18.84	<0.0001	264.35	60.94
Minimum base area (cm^2^)	847668	1	7.61	0.007	−62.05	22.49
Maximum base area (cm^2^)	1242860	1	11.16	0.0012	−37.67	11.28
Species	57274564	20	2.57	0.0012		
Error	10354141	93				

## Discussion

The main findings of this study of the relationship between clutch size and nest size are that most populations and species of both hole and open nesters show increasing clutch size with increasing nest size, with a general increase that varied within and among species. This relationship did not differ between hole and open nesters.

Clutch size was directly affected by nest size, as shown by Löhrl’s (1973, 1980) experiments that manipulated the floor area of nest boxes after clutch initiation and thereby produced significant differences in clutch size. Here, we have extended Löhrl’s findings by showing that the relationship between clutch size and nest size was similar when females could or could not choose a specific box size. Löhrl (1973, 1980) used boxes with base area of 64 or 314 cm^2^, or almost a fivefold difference. However, this difference is similar to natural variation in base area in the great tit (van Balen et al. 1982). This implies that differential access to preferred boxes is not the main cause of bias in estimates of the relationship between clutch size and nest base area. Soler et al. (2001) have shown for magpies *Pica pica* that experimental reduction of the size of the roof reduced clutch size. Given that females responded to a change in the size of the part of the nest that does not hold the eggs and the nestlings, we can conclude that it is nest size and nest building, but not nest cup size that is driving clutch size decisions of the female. Most analyses of the relationship between clutch size and our proxy of nest size that we have reported in Table 1 were statistically significant and slopes were in many cases positive with overlapping 95% confidence intervals among experiments and data sets and for open and hole nesters. Interestingly, the plasticity of the relationship between clutch size and nest size was similar in open and hole-nesting species. Among great tits, there was evidence of one standard deviation in nest base area equaling an increase in clutch size by 1.1–3.3 eggs, which is a considerable change given a modal clutch size of 8.69 eggs in 1453 population-year estimates. We suggest that in natural habitats cavities may differ substantially in size and shape, resulting in the evolution of phenotypic plasticity of clutch size in relation to nest size, although this observation does not explain the difference in reaction norms among hole-nesting species. More recently, competition for nest holes of an optimal size in man-managed forests, where older, larger, and dead trees are regularly removed, may lead to a scarcity of natural cavities for breeding birds (Sandström 1992; Carlson et al. 1998; Sánchez et al. 2007) and may have contributed to the evolution of this extreme reaction norm. We did find evidence of statistical heterogeneity among four secondary hole-nesting species and among more than 17,000 nests belonging to 21 species of birds. Among the four common species of secondary hole-nesting species, great tits showed a significant positive relationship, while blue tits and the two *Ficedula* flycatchers showed no significant relationship (Table 1; Møller et al. 2014). In addition, the 95% CI for great tit did not overlap with the 95% CI for blue tit or the two flycatcher species. Interestingly, intraspecific studies on the relationship between nest size and reproductive success have shown a significant relationship in great tits (Alabrudzinska et al. 2003; Álvarez and Barba 2008), but not in blue tits (Lambrechts et al. 2012). As we stated in the Introduction, the relationship between clutch size and nest size may arise as a consequence of female choice of an appropriately sized box in relation to the future clutch size of the female. In fact, nest volume was positively correlated with nest base area as shown in five studies of two species of hole nesters and two species of open nesters. However, it seems unlikely that this relationship is caused by nest site choice as nest cups are usually considerably smaller than the base area of nest boxes, and the relationship between clutch size and nest size did not differ significantly between open and hole nesters.

In addition, the relationship between clutch size and nest size could arise as the reaction norm of individual females to their nest sites. Löhrl’s (1980) study of great tits and our re-analysis of his data clearly show that the latter mechanism is at work. Given that the slope of the relationship is stronger for the reaction of the female rather to altered box size than the choice of box size by the female, this implies that the former mechanism may be sufficient for explaining the patterns of phenotypic variation. In addition, an analysis of all studies with two or more nest or box sizes showed significant heterogeneity in slopes among the 21 species of birds including the three open nesting species, but also significant differences in variance within species (Table 2). However, the relationship did not differ significantly between open nesting and hole-nesting species. Given that hole nesting is a derived character, we can conclude that the relationship between nest size and clutch size evolved before hole nesting, justifying the inclusion of open-nesting species in the present study.

Slagsvold (1987) provided the most exhaustive list of hypotheses accounting for the association between clutch size and nest base area within species, using extensive experiments on the pied flycatcher. The seven hypotheses listed by Slagsvold can basically be grouped into hypotheses concerning energetics, nest predation, and sexual selection. Here, we have documented differences in the relationship between clutch size and nest size as reflected by the slope among four species of secondary hole nesters and among 21 species of birds, for which we had data, suggesting that the interspecific differences are real. The phylogenetic signal in these data must be weak at best given that species only accounted for a couple of per cent of the variance. Given that an increase in nest floor area by 100 cm^2^ is equivalent to an additional 0.36 eggs in the 21 species investigated or 1.25 eggs in the four secondary hole nesters, these effects cannot be considered negligible. An increase in floor area of 100 cm^2^ is similar to standard deviations of 5-165 cm^2^ in ten hole-nesting species in natural holes (van Balen et al. 1982, Table 14). We found no significant effects of latitude and longitude or a number of other variables predicted to affect this relationship between clutch size and nest size.

The analyses reported here suggest that the reaction norm of the relationship between clutch size and nest size is similar in nest boxes and in natural holes, because the effect of nest type (nest box or natural hole) did not enter the statistical analyses as a significant predictor. This conclusion is comforting because it suggests that scientists working with nest box breeding birds are studying natural phenomena within a single context (Møller 1989; Lambrechts et al. 2010). However, these findings also suggest that comparison of observations among nest box sizes within or among populations should be made with care. We found evidence that in particular the minimum size of nests/boxes used, but less so the maximum size was significantly related to slope estimates, as shown for the analysis of studies with two or more sizes of nests/boxes used. This greater difference for the minimum size of nest boxes used than for the maximum size of nest boxes used suggests that the reaction norm is increasing for the smallest nests/boxes, but less so for the largest nests/boxes for the range of sizes investigated so far. One way forward would be to estimate the relationship between clutch size and nest size experimentally using a range of nest base areas from 25 to 500 cm^2^, while experimentally manipulating clutch size, with replicates from North Africa to Northern Scandinavia. If hyperthermia is the underlying selective factor accounting for the relationship between clutch size and nest size, we should expect stronger negative effects in a small nest box treatment with an enlarged clutch size at low latitudes.

In conclusion, we have shown in this exhaustive analysis of the relationship between clutch size and base area of nests that such a relationship is common across populations and species with differences in effects among populations within species and among species. The findings suggest that scientists by choosing particular nest box sizes affect clutch size and hence potentially reproductive success in a species-specific manner (Møller et al. 2014). These findings may be of general importance for nest building taxa of insects, fish and mammals. However, such tests must await future studies of other taxa since no comparable studies are currently available.

## Supporting Information

Table S1 Summary information on studies of the relationship between clutch size and nest size in different species of birds.
