# Supplementary material for: Variation in clutch size in relation to nest size in birds
Source: Ecol Evol. 2014 Sep 2;4(18):3583–95. doi: 10.1002/ece3.1189 (PMC4224533; doi:10.1002/ece3.1189)
Supplement: Table S1 — Summary information on studies of the relationship between clutch size and nest size in different species of birds. [file ece30004-3583-sd1.doc]

**Electronic Supplementary Material Table S1.** Summary information on studies of the relationship between clutch size and nest size in different species of birds. Species, locality, latitude (ºN), longitude (ºE), slope, SE, F statistic, probability P, sample size N, nest box (yes, no), body mass (g), nest building (yes, no) and reference. See Materials and methods for further details.

| Species | Locality | Latitude | Longitude | Slope | SE | Test statistic | *P* | *N* | Nest box | Body mass (g) | Nest building | Reference |
| --- | --- | --- | --- | --- | --- | --- | --- | --- | --- | --- | --- | --- |
| *Aegolius funereus* | Kauhava | 63 | 23 | 0.00372 | 0.00094 | 15.60 | 0.01 | 215 | Yes | 143.00 | No | Valkema and Korpimäki (1999) |
| *Cercotrichas galactotes* | Spain | 37 | -2 | -0.00041 | 0.00292 | 0.48 | 0.89 | 43 | No | 22.15 | Yes | Palomino et al. (1998) |
| *Colaptes auratus* | Canada | 52 | -122 | 0.00041 | 0.00360 |  |  | 121 | No | 132.00 | Yes | Wiebe & Swift (2001) |
| *Cyanistes caeruleus* | Cantabrian Mountains | 42 | -5 | 0.01418 | 0.02337 |  |  | 62 | Yes | 11.75 | No | H. Robles et al. (2011) |
| *Cyanistes caeruleus* | Finestres | 42 | 2 | 0.01250 | 0.01162 |  |  | 7 | Yes | 11.75 | No | J. Camprodon pers. comm. |
| *Cyanistes caeruleus* | Foretd'Orient | 48 | 4 | 0.01606 | 0.00409 |  |  | 267 | Yes | 11.75 | No | C. Biard pers. comm. |
| *Cyanistes caeruleus* | Gotland-TU | 57 | 18 | 0.02175 | 0.10351 |  |  | 60 | Yes | 11.75 | No | L. Gustafsson pers. comm. |
| *Cyanistes caeruleus* | Liesbos | 53 | 0 | 0.02277 | 0.01725 |  |  | 65 | Yes | 11.75 | No | H. Van Balen (1984) |
| *Cyanistes caeruleus* | Lodz. Lagiewniki Forest | 52 | 19 | -0.00365 | 0.00393 |  |  | 168 | Yes | 11.75 | No | J. Banbura pers. comm. |
| *Cyanistes caeruleus* | Marley Woods | 51 | -1 | 0.01399 | 0.01400 |  |  | 25 | No | 11.75 | No | East & Perrins (1988) |
| *Cyanistes caeruleus* | Prepirineu | 42 | 2 | 0.00725 | 0.01025 |  |  | 8 | Yes | 11.75 | No | J. Camprodon pers. comm. |
| *Cyanistes caeruleus* | Montseny | 42 | 2 | 0.03870 | 0.01674 |  |  | 4 | Yes | 11.75 | No | J. Camprodon pers. comm. |
| *Cyanistes caeruleus* | Ruskeasuo-Maunula. Helsinki | 60 | 24 | -0.00341 | 0.00622 |  |  | 97 | Yes | 11.75 | No | Solonen (2001) |
| *Cyanistes caeruleus* | Stenbrohult | 56 | 14 | -0.03034 | 0.00949 |  |  | 19 | Yes | 11.75 | No | S. G. Nilsson et al. pers. comm. |
| *Cyanistes caeruleus* | Wilrijk | 51 | 4 | 0.00843 | 0.00657 |  |  | 246 | Yes | 11.75 | No | M. Eens et al. pers. comm. |
| *Cyanistes caeruleus* | Near Dijon | 47 | 5 | -0.00370 | 0.00621 |  |  | 36 | Yes | 11.75 | No | B. Faivre et al. pers. comm. |
| *Cyanistes caeruleus* | Bialowieza | 53 | 24 | -0.00160 | 0.00270 | 0.34 | 0.56 | 123 | No | 11.75 | No | Wesolowski &Rowinski (2012) |
| *Falco sparverius* | Besnard Lake | 55 | -187 | 0.00000 |  |  |  | 87 | Yes | 115.50 | No | Bortolotti (1994) |
| *Falco tinnunculus* | Kauhava | 63 | 23 | 0.00034 | 0.00034 |  | 0.50 | 178 | Yes | 174.50 | No | Korpimäki (1985) |
| *Ficedula albicollis* | Bialowieza | 53 | 24 | 0.00101 | 0.00062 | 2.6842 | 0.1022 | 391 | No | 13.40 | No | Czeszczewik & Walankiewicz (2003) |
| *Ficedula albicollis* | Gotland-FA | 57 | 18 | 0.00349 | 0.00492 |  |  | 634 | Yes | 13.40 | No | L. Gustafsson pers. comm. |
| *Ficedula albicollis* | Gotland-GR | 57 | 18 | 0.00616 | 0.00351 |  |  | 772 | Yes | 13.40 | No | L. Gustafsson pers. comm. |
| *Ficedula hypoleuca* | Tärnsjö | 60 | 16 | 0.00600 |  | tau = 0.130 | 0.01 | 164 | Yes | 14.35 | No | Karlsson & Nilsson (1977)) |
| *Ficedula hypoleuca* | Tärnsjö | 60 | 16 | 0.01000 |  | tau = 0.194 | 0.001 | 145 | Yes | 14.35 | No | Karlsson & Nilsson (1977)) |
| *Ficedula hypoleuca* | Tärnsjö | 60 | 16 | 0.00400 |  | tau = 0.014 | 0.1 | 100 | Yes | 14.35 | No | Karlsson & Nilsson (1977)) |
| *Ficedula hypoleuca* | Stenbrohult | 56 | 14 | 0.00500 |  | tau = 0.217 | 0.09 | 20 | Yes | 14.35 | No | Karlsson & Nilsson (1977)) |
| *Ficedula hypoleuca* | Stenbrohult | 56 | 14 | 0.01900 |  | tau = 0.012 | 0.012 | 16 | Yes | 14.35 | No | Karlsson & Nilsson (1977)) |
| *Ficedula hypoleuca* | Uppsala | 60 | 18 | 0.00015 | 0.00067 | 0.0501 | 0.8235 | 66 | Yes | 14.35 | No | Alatalo et al. (1988) |
| *Ficedula hypoleuca* | Bialowieza | 53 | 24 | -0.00020 | 0.00027 | 0.5567 | 0.46 | 37 | Yes | 14.35 | No | Czeszczewik & Walankiewicz (2003) |
| *Ficedula hypoleuca* | Dæli, Bærum | 60 | 11 | 0.00149 | 0.00513 |  |  | 48 | Yes | 14.35 | No | T. Slagsvold pers. comm. |
| *Ficedula hypoleuca* | ?akiai | 55 | 23 | -0.01028 | 0.00740 |  |  | 371 | Yes | 14.35 | No | Ju?kaitis R.. pers. com. |
| *Ficedula hypoleuca* | S?kocin | 52 | 21 | -0.00142 | 0.00558 |  |  | 56 | Yes | 14.35 | No | Mazgajski T.. pers com. |
| *Ficedula hypoleuca* | Stenbrohult | 56 | 14 | 0.00713 | 0.00253 |  |  | 120 | Yes | 14.35 | No | S. G. Nilsson et al. pers. comm. |
| *Ficedula hypoleuca* | Szwalewo Forest | 56 | 14 | -0.00353 | 0.00448 |  |  | 253 | Yes | 14.35 | No | W. Kania pers. comm. |
| *Ficedula hypoleuca* | Vistula Spit | 54 | 19 | -0.00340 | 0.00392 |  |  | 135 | Yes | 14.35 | No | W. Kania pers. comm. |
| *Ficedula hypoleuca* | Chrzelice | 50 | 17 | -0.01553 | 0.00600 | 6.6971 | 0.0111 | 105 | Yes | 14.35 | No | Graczyk (1967) |
| *Ficedula hypoleuca* | Kilpisjärvi | 69 | 21 | -0.00240 | 0.00279 |  |  | 1050 | Yes | 14.35 | No | Järvinen (1983) |
| *Ficedula hypoleuca* | Konnevesi | 62 | 26 | 0.00805 | 0.00902 |  |  | 171 | Yes | 14.35 | No | Hemborg (1998), M. Mönkkönen pers. comm. |
| *Ficedula hypoleuca* | Kraipe | 65 | 16 | 0.00204 | 0.01284 | 0.0252 | 0.884 | 244 | Yes | 14.35 | No | Svensson (1987) |
| *Ficedula hypoleuca* | Nagshead Plantation | 51 | -2 | -0.00275 | 0.00057 |  |  | 1817 | Yes | 14.35 | No | A. Goodenough pers. comm., P. H. Harvey et al. (1988) |
| *Ficedula hypoleuca* | Oulu | 65 | 25 | 0.01664 | 0.00201 | 68.77 | 0.0764 | 198 | Yes | 14.35 | No | Forsman et al. (2011), O. Loukola pers. comm. |
| *Ficedula hypoleuca* | Trondheim, Ler West | 63 | 10 | 0.00333 | 0.00249 |  |  | 71 | Yes | 14.35 | No | Slagsvold (1987) |
| *Hirundo rustica* | Kraghede | 57 | 10 | 0.00048 | 0.00082 | 0.35 | 0.56 | 58 | No | 19.10 | Yes | A. P. Møller unpublished data |
| *Hirundo rustica* | Kraghede | 57 | 10 | 0.00260 | 0.00280 | 0.86 | 0.36 | 46 | No | 19.10 | Yes | A. P. Møller unpublished data |
| *Hirundo rustica* | Kraghede | 57 | 10 | 0.00620 | 0.00290 | 4.50 | 0.049 | 19 | No | 19.10 | Yes | A. P. Møller unpublished data |
| *Hirundo rustica* | Kraghede | 57 | 10 | 0.00760 | 0.00310 | 6.05 | 0.034 | 12 | No | 19.10 | Yes | A. P. Møller unpublished data |
| *Hirundo rustica* | Kraghede | 57 | 10 | 0.00110 | 0.00330 | 0.12 | 0.13 | 28 | No | 19.10 | Yes | A. P. Møller unpublished data |
| *Hirundo rustica* | Kraghede | 57 | 10 | 0.00180 | 0.00240 | 0.58 | 0.45 | 53 | No | 19.10 | Yes | A. P. Møller unpublished data |
| *Hirundo rustica* | Kraghede | 57 | 10 | 0.00430 | 0.00240 | 3.19 | 0.081 | 46 | No | 19.10 | Yes | A. P. Møller unpublished data |
| *Hirundo rustica* | Kraghede | 57 | 10 | 0.00200 | 0.00240 | 0.68 | 0.42 | 53 | No | 19.10 | Yes | A. P. Møller unpublished data |
| *Hirundo rustica* | Kraghede | 57 | 10 | 0.00420 | 0.00240 | 3.07 | 0.09 | 47 | No | 19.10 | Yes | A. P. Møller unpublished data |
| *Hirundo rustica* | Kraghede | 57 | 10 | 0.00030 | 0.00600 | 0.003 | 0.96 | 25 | No | 19.10 | Yes | A. P. Møller unpublished data |
| *Hirundo rustica* | Kraghede | 57 | 10 | 0.02250 | 0.00780 | 8.29 | 0.012 | 17 | No | 19.10 | Yes | A. P. Møller unpublished data |
| *Hirundo rustica* | Kraghede | 57 | 10 | -0.00020 | 0.00470 | 0.002 | 0.97 | 29 | No | 19.10 | Yes | A. P. Møller unpublished data |
| *Hirundo rustica* | Kraghede | 57 | 10 | 0.01600 | 0.00560 | 8.31 | 0.0054 | 64 | No | 19.10 | Yes | A. P. Møller unpublished data |
| *Hirundo rustica* | Kraghede | 57 | 10 | 0.00550 | 0.00460 | 1.44 | 0.24 | 47 | No | 19.10 | Yes | A. P. Møller unpublished data |
| *Hirundo rustica* | Kraghede | 57 | 10 | 0.01380 | 0.00630 | 4.83 | 0.033 | 51 | No | 19.10 | Yes | A. P. Møller unpublished data |
| *Hirundo rustica* | Kraghede | 57 | 10 | 0.00340 | 0.00480 | 1.23 | 0.23 | 101 | No | 19.10 | Yes | A. P. Møller unpublished data |
| *Hirundo rustica* | Kraghede | 57 | 10 | 0.00970 | 0.00550 | 3.06 | 0.084 | 91 | No | 19.10 | Yes | A. P. Møller unpublished data |
| *Hirundo rustica* | Kraghede | 57 | 10 | 0.00090 | 0.00550 | 0.024 | 0.88 | 67 | No | 19.10 | Yes | A. P. Møller unpublished data |
| *Hirundo rustica* | Kraghede | 57 | 10 | 0.01770 | 0.00420 | 18.13 | <0.0001 | 86 | No | 19.10 | Yes | A. P. Møller unpublished data |
| *Hirundo rustica* | Kraghede | 57 | 10 | 0.00200 | 0.00510 | 0.15 | 0.70 | 133 | No | 19.10 | Yes | A. P. Møller unpublished data |
| *Hirundo rustica* | Kraghede | 57 | 10 | -0.00001 | 0.00005 | 0.08 | 0.78 | 184 | No | 19.10 | Yes | A. P. Møller unpublished data |
| *Hirundo rustica* | Kraghede | 57 | 10 | 0.00002 | 0.00004 | 0.23 | 0.63 | 217 | No | 19.10 | Yes | A. P. Møller unpublished data |
| *Hirundo rustica* | Kraghede | 57 | 10 | 0.00005 | 0.00008 | 0.29 | 0.59 | 185 | No | 19.10 | Yes | A. P. Møller unpublished data |
| *Hirundo rustica* | Kraghede | 57 | 10 | 0.00003 | 0.00006 | 0.18 | 0.68 | 159 | No | 19.10 | Yes | A. P. Møller unpublished data |
| *Hirundo rustica* | Kraghede | 57 | 10 | 0.00001 | 0.00007 | 0.004 | 0.95 | 105 | No | 19.10 | Yes | A. P. Møller unpublished data |
| *Myiarchus cinerascens* | San Joaquin Experimental Range | 37 | -120 | 0.00700 |  |  | 0.232 | 33 | Yes | 27.20 | No | Purcell et al. (1997) |
| *Parus inornatus* | San Joaquin Experimental Range | 37 | -120 | 0.00200 |  |  | 0.148 | 111 | Yes | 17.50 | No | Purcell et al. (1997) |
| *Parus major* | Germany | 49 | 8 | 0.00545 | 0.00150 | 12.78 | < 0.0001 | 29 | Yes | 18.50 | No | Löhrl (1973) |
| *Parus major* | Germany | 49 | 8 | 0.01258 | 0.00302 | 17.4 | 0.0005 | 22 | Yes | 18.50 | No | Löhrl (1973) |
| *Parus major* | Tärnsjö | 60 | 16 | 0.02800 |  | tau = 0.208 | 0.01 | 66 | Yes | 18.50 | No | Karlsson & Nilsson (1977)) |
| *Parus major* | Tärnsjö | 60 | 16 | 0.02700 |  | tau = 0.256 | 0.001 | 97 | Yes | 18.50 | No | Karlsson & Nilsson (1977)) |
| *Parus major* | Tärnsjö | 60 | 16 | 0.02800 |  | tau = 0.304 | 0.001 | 66 | Yes | 18.50 | No | Karlsson & Nilsson (1977)) |
| *Parus major* | Stenbrohult | 56 | 14 | 0.03400 |  | tau = 0.423 | 0.001 | 27 | Yes | 18.50 | No | Karlsson & Nilsson (1977)) |
| *Parus major* | Germany | 49 | 8 | 0.01320 | 0.00518 | 6.48 | 0.0203 | 20 | Yes | 18.50 | No | Löhrl (1980) |
| *Parus major* | Bern Bremer | 46 | 7 | 0.01342 | 0.00184 |  |  | 562 | Yes | 18.50 | No | H. Richner |
| *Parus major* | Cantabrian Mountains | 42 | -5 | -0.02491 | 0.02821 |  |  | 39 | Yes | 18.50 | No | H. Robles et al. (2011) |
| *Parus major* | Foretd'Orient | 48 | 4 | 0.03656 | 0.00447 |  |  | 209 | Yes | 18.50 | No | C. Biard pers. comm. |
| *Parus major* | Kauhava | 63 | 23 | 0.00176 | 0.00461 |  |  | 40 | Yes | 18.50 | No | E. Korpimäki pers. comm. |
| *Parus major* | Liesbos | 53 | 0 | 0.02416 | 0.00088 |  |  | 147 | Yes | 18.50 | No | H. Van Balen (1984) |
| *Parus major* | Lodz. Lagiewniki Forest | 52 | 19 | 0.02140 | 0.00612 |  |  | 114 | Yes | 18.50 | No | J. Banbura pers. comm. |
| *Parus major* | Marley Woods | 51 | -1 | 0.01639 | 0.03115 |  |  | 13 | No | 18.50 | No | East & Perrins (1988) |
| *Parus major* | Oberrheinebene | 49 | 8 | 0.00830 | 0.00106 |  |  | 57 | Yes | 18.50 | No | H. Löhrl (1973, 1980) |
| *Parus major* | Prepirineu | 42 | 2 | -0.03333 | 0.11501 |  |  | 35 | Yes | 18.50 | No | J. Camprodon pers. comm. |
| *Parus major* | Montseny | 42 | 2 | 0.00000 | 0.15430 |  |  | 21 | Yes | 18.50 | No | J. Camprodon pers. comm. |
| *Parus major* | Ruskeasuo-Maunula. Helsinki | 60 | 24 | 0.01316 | 0.00525 |  |  | 323 | Yes | 18.50 | No | Solonen (2001) |
| *Parus major* | ?akiai | 55 | 23 | -0.02664 | 0.00603 |  |  | 377 | Yes | 18.50 | No | Ju?kaitis R.. pers. com. |
| *Parus major* | Stenbrohult | 56 | 14 | 0.04713 | 0.01676 |  |  | 75 | Yes | 18.50 | No | S. G. Nilsson et al. pers. comm. |
| *Parus major* | Ville-Montpellier | 43 | 3 | 0.00707 | 0.00188 |  |  | 148 | Yes | 18.50 | No | A. Gregoire et al. pers. comm. |
| *Parus major* | Wilrijk | 51 | 4 | 0.00474 | 0.00205 |  |  | 733 | Yes | 18.50 | No | M. Eens et al. pers. comm. |
| *Parus major* | Chrzelice | 50 | 17 | 0.00719 | 0.01285 | 0.31 | 0.58 | 33 | Yes | 18.50 | No | Graczyk (1967) |
| *Parus major* | Chrzelice | 50 | 17 | 0.01168 | 0.01114 | 1.0988 | 0.2983 | 68 | Yes | 18.50 | No | Graczyk (1967) |
| *Parus major* | Barcelona | 41 | 2 | 0.00243 | 0.00109 |  |  | 452 | Yes | 18.50 | No | J. C. Senar pers. comm. |
| *Parus major* | Burano | 42 | 11 | 0.00043 | 0.00045 | 0.9265 | 0.5121 | 19 | Yes | 18.50 | No | A. Sorace pers. comm. |
| *Parus major* | Haga, Sinober | 60 | 11 | 0.02000 | 0.00525 |  |  | 12 | Yes | 18.50 | No | Slagsvold & Amundsen (1992) |
| *Parus major* | Kilpisjärvi | 69 | 21 | -0.00620 | 0.00332 |  |  | 147 | Yes | 18.50 | No | Järvinen (1983) |
| *Parus major* | Moshav Ram On-Rural | 32 | 35 | 0.00000 | 0.00174 |  |  | 37 | Yes | 18.50 | No | M. Charter pers. comm. |
| *Parus major* | Near Dijon | 47 | 5 | 0.01111 | 0.00586 |  |  | 40 | Yes | 18.50 | No | B. Faivre et al. pers. comm. |
| *Parus major* | Trondheim, Ler West | 63 | 10 | 0.00686 | 0.00727 |  |  | 22 | Yes | 18.50 | No | T. Slagsvold pers. comm. |
| *Parus major* | Italy | 45 | 12 | 0.00210 |  |  |  | 31 | Yes | 18.50 | No | Sorace & Carere (1996) |
| *Parus montanus* | Germany2 |  |  | 0.07200 |  | tau = 0.659 | 0.001 | 28 | No | 11.65 | Yes | Ludescher (1973) |
| *Parus palustris* | Germany2 |  |  | 0.11700 |  | tau = 0.632 | 0.001 | 21 | No | 11.90 | Yes | Ludescher (1973) |
| *Parus palustris* | Bialowieza | 53 | 24 | 0.00490 | 0.00150 |  | 0.0014 | 366 | No | 11.90 | Yes | Wesolowski (2003) |
| *Passer domesticus* | Homewood, IL | 41 | -87 | 0.00365 | 0.00114 | 10.19 | 0.0044 | 23 | Yes | 30.35 | No | Lowther (2013) |
| *Passer italiae* | Italy | 45 | 12 | 0.00093 |  |  |  | 32 | Yes | 30.35 | No | Sorace & Carere (1996) |
| *Sialia mexicana* | San Joaquin Experimental Range | 37 | -120 | 0.01100 |  |  | 0.709 | 47 | Yes | 28.05 | No | Purcell et al. (1997) |
| *Sitta europaea* | Magadan | 60 | 151 | -0.00781 | 0.00600 | 1.69 | 0.2071 | 25 | No | 23.90 | No | Pravosudov (1995) |
| *Sturnus vulgaris* | Krankesjön | 56 | 13 | -0.00020 |  | tau = -0.062 | 0.10 | 28 | Yes | 80.50 | No | Karlsson & Nilsson (1977)) |
| *Sturnus vulgaris* | Krankesjön | 56 | 13 | 0.00070 |  | tau = -0.015 | 0.10 | 33 | Yes | 80.50 | No | Karlsson & Nilsson (1977)) |
| *Sturnus vulgaris* | Queen's University Field Station | 50 | 9 | 0.00365 | 0.00076 | 23.33 | < 0.0001 | 130 | Yes | 80.50 | No | Trillmich & Hudde (1984) |
| *Sturnus vulgaris* | New Zealand | -39 | 176 | -0.00029 | 0.00043 | 0.044 | 0.52 | 90 | Yes | 80.50 | No | Mooed & Dawson (1979) |
| *Sturnus vulgaris* | Buissonville | 50 | 5 | 0.00260 |  |  |  | 1540 | Yes | 80.50 | No | Clobert & Berthet (1983) |
| *Tachycineta bicolor* | Queen's University Field Station | 45 | -76 | 0.00470 | 0.00430 |  |  | 27 | Yes | 20.10 | No | Rendell & Robertson (1993) |
| *Tachycineta bicolor* | Queen's University Field Station | 45 | -76 | 0.00410 | 0.00240 |  |  | 83 | Yes | 20.10 | No | Rendell & Robertson (1993) |
| *Troglodytes aedon* | San Joaquin Experimental Range | 37 | -120 | 0.00200 |  |  | 0.815 | 36 | Yes | 10.90 | No | Purcell et al. (1997) |
